# Within-Person Fluctuations in Objective Smartphone Use and Emotional Processes During Adolescence: An Intensive Longitudinal Study

**DOI:** 10.1007/s42761-024-00247-z

**Published:** 2024-07-24

**Authors:** Alexandra M. Rodman, Jason A. Burns, Grace K. Cotter, Yuri-Grace B. Ohashi, Rachael K. Rich, Katie A. McLaughlin

**Affiliations:** 1 Department of Psychology, Northeastern University, Boston, MA USA; 2 Department of Psychology, Harvard University, Cambridge, MA USA; 3 The Ballmer Institute, University of Oregon, Portland, OR USA

**Keywords:** Adolescence, Social media, Smartphone, Positive mood, Negative mood, Longitudinal

## Abstract

Since the advent of smartphones, peer interactions over digital platforms have become a primary mode of socializing among adolescents. Despite the rapid rise in digital social activity, it remains unclear how this dramatic shift has impacted adolescent social and emotional experiences. In an intensive, longitudinal design (*N* = 26, *n* = 206 monthly observations for up to 12 months, 12–17 years), we used digital phenotyping methods to objectively measure within-person fluctuations in smartphone use (screen time, pickups, notifications) across different categories (social media, communication, entertainment, games) and examined their prospective, bidirectional associations with positive and negative mood. Bayesian hierarchical models showed that when adolescents reported better mood than usual, they subsequently spent more time on communication apps and launched social media and communication apps upon pickup less often. Meanwhile, when adolescents used entertainment apps more than usual, they subsequently reported improved mood. These preliminary findings suggest a pattern where fluctuations in mood relate to subsequent changes in smartphone use that are primarily social, whereas the fluctuations in smartphone use relating to subsequent changes in mood were primarily entertainment-related. We found little evidence that within-person fluctuations in screen time or social media use were associated with increases in negative mood, as frequently theorized. These findings highlight the importance of disentangling the distinct components of smartphone use that relate to affective processes and examining their bidirectional, prospective relationships over time, due to the possibility of differential outcomes. This work is a necessary first step in identifying targets for intervention efforts promoting resilience and wellbeing during adolescence.

**Supplementary Information:**

The online version contains supplementary material available at 10.1007/s42761-024-00247-z.

Adolescence is a unique period of social sensitivity (Somerville, 2013) when the importance of peer relationships and belonging increases (Brown, 1990; Somerville, 2013). Social interactions through smartphones have become a primary mode of connection among adolescents (Lenhart et al., 2010), becoming completely integrated with everyday life, as 95% of adolescents have a smartphone and are online frequently throughout the day (Pew Research Center, 2018; Rideout et al., 2022; Vogels et al., 2022). Yet, it remains unclear how this dramatic shift in the adolescent social landscape has impacted wellbeing. While socializing through smartphones can strengthen relationships, they may also increase exposure to negative social experiences (e.g., rejection, cyberbullying). Research examining this direct link has been inconclusive (Odgers & Jensen, 2020; Valkenburg et al., 2022) and many in the field have called for improved measurements to help clarify these relationships (Beyens et al., 2021; Kross et al., 2021; Orben & Blakemore, 2023; Valkenburg, 2022). Meanwhile, reported affect is more frequent, intense, and volatile during adolescence (Guyer et al., 2016; Steinberg, 2005) and involves reported decreases in positive affect and increases in negative affect (Abitante et al., 2022; Griffith et al., 2021; Grisanzio et al., 2023). Given the rapid socioemotional development and sharp rise in mental health problems that occur during adolescence (Blakemore & Mills, 2014; Crone & Dahl, 2012; Paus et al., 2008), it is important to understand how affective processes are related to smartphone use during adolescence (Orben et al., 2024). The current study leverages objective measures of smartphone use in an intensive, longitudinal (i.e., within-person) study that examines more granular features of smartphone use and its relation to adolescent emotional experience, a critical marker of adolescent mental health and wellbeing.

Extant research has revealed that the impact of smartphone use on adolescent outcomes is complex, varied, and context-dependent. Research in this area has recently accelerated, but consensus remains elusive (Appel et al., 2020; Hamilton et al., 2022; Jensen et al., 2019; Johannes et al., 2021; Valkenburg et al., 2022). On the one hand, youth who reported greater smartphone use showed either no change or improved associations with loneliness, strength of relationships, social support, and internalizing symptoms (Anto et al., 2023; Coyne et al., 2020; George et al., 2018; Jensen et al., 2019; Padilla‐Walker et al., 2012; Roser et al., 2016; Steinsbekk et al., 2023; Thomée et al., 2011). Work using objective, longitudinal data has yielded similarly positive or benign outcomes (Maftei et al., 2022; Marciano et al., 2022). On the other hand, reported smartphone use has been associated with worse emotional health, self-esteem, and wellbeing (Bennett et al., 2020; McNamee et al., 2021). Total time spent online and particularly high volumes of digital communication have also been correlated with worse self-harm, internalizing symptoms, and daily functioning (Coyne et al., 2019; McAllister et al., 2021). These discrepant findings could be clarified by examining intermediary factors (thoughts and emotions related to smartphone use) that shape adolescent wellbeing. Posting and interacting with peers on social media has been related to enhanced self-expression, affirmation, and positive affect (James et al., 2023; Karsay et al., 2022). However, both self-reported and objective smartphone use have been associated with social comparison and negative mood states (Dreier et al., 2024; Engeln et al., 2020; Nereim et al., 2022; Ren et al., 2023; Sequeira et al., 2020), that, in turn, modulate clinical risk (Nesi et al., 2022; Nick et al., 2022).

Ultimately, the extant literature on adolescent smartphone use exhibits considerable heterogeneity, showing associations with both positive and negative outcomes. Inconsistencies may be partially explained by the self-reported and cross-sectional nature of the majority of early research in this area, much of which combines all digital media use into a single composite rather than examining more granular types of use (Ellis, 2019; Kross et al., 2021). The current study aims to overcome these challenges by leveraging objective measures of smartphone use in an intensive, longitudinal design that examines granular features of smartphone use and its relation to adolescent mood. Adolescents use smartphones in a multitude of ways (Toh et al., 2019; Twenge & Farley, 2021), where associations with wellbeing vary by specific categories of apps (David et al., 2018) or types of smartphone use (Marciano et al., 2022; Oulasvirta et al., 2012; Rozgonjuk et al., 2018). Social (e.g., social media, communication) and non-social (e.g., entertainment, games) smartphone use can shape adolescent development (Allaby & Shannon, 2020) and are differentially associated with anxiety and depression (Elhai et al., 2017). While different phone metrics—like screen time (duration), pickups (app launched after pickup), and notifications—are related, they reflect fundamentally different behaviors and experiences (i.e., controllability) that are differentially related to positive and negative outcomes (Dreier et al., 2024; Kanjo et al., 2017; Prinstein et al., 2020; Saeb et al., 2015; Toh et al., 2023). Though screen time has been a leading metric of phone use in many early and influential studies, an emerging literature examining other metrics of phone use, such as pickups and notifications, has developed. Frequency of pickups and notifications have both been associated with positive and negative affect (Dreier et al., 2024; Kanjo et al., 2017; Saeb et al., 2015), as well as depressed mood and distraction (Rozgonjuk et al., 2018; Stothart et al., 2015; Toh et al., 2023; Upshaw et al., 2022). Distinguishing between social and non-social use may clarify these inconsistent findings (Kanjo et al., 2017). Thus, better characterization of these metrics and related psychological experiences is critical to informing best practices for smartphone management to enhance wellbeing.

We examine screen time, pickups, and notifications across five categories: overall use, social media, communication, games, and entertainment. We examine the bidirectional, prospective relationships between smartphone use and mood, a key precursor of wellbeing (Watson et al., 1988), at the within-person, monthly level for up to 12 months. This intensive, longitudinal design can capture the substantial within-person variability evident in social and affective processes (Coppersmith et al., 2019; Dewald-Kaufmann et al., 2021; Rodman et al., 2021), and characterize how these relationships unfold over time. Drawing from our previous work (Rodman et al., 2021), we expect that social media use and communication will have prospective and bidirectional associations with negative mood. Due to the limited study of other smartphone use categories and metrics, such as pickups and notifications, the examination of their relationships to mood is exploratory.

## Method

All data and code are made available on Open Science Framework at: https://osf.io/t36wd/.

### Participants

Participants were drawn from an ongoing intensive longitudinal parent study involving monthly assessments for 1 year. The current sample included 26 adolescents (*n* = 206 monthly observations) aged 12–17 years (*M*_age_ = 15.18 years, *SD*_age_ = 1.12 years, 42% female-identifying; see Table 1). Our study was well-powered to examine within-person associations between smartphone use and positive and negative mood over time, with sufficient power (> 80%) to detect small within-person effects (*ß* = 0.12). See *Supplementary Information* and Figure S3 for more information on the simulated power analysis approach. Participants were recruited from schools, libraries, public transportation, and other public spaces in the general community in the greater Boston, MA area and participated in the current study between March 2021 and May 2022. Inclusion criteria required being aged 12–17 years old, possession of a smartphone with a data plan, and English fluency. Participants were excluded from the parent study based on the following criteria: presence of pervasive developmental disorders (e.g., autism), MRI scan ineligibility (e.g., metal implants, metal braces, claustrophobia, pregnancy), psychiatric medication use, active safety concerns, and inability to attend 12 study sessions at Harvard University. The community-based sample was broadly representative of diverse racial and ethnic backgrounds, with 42% of participants identifying as White, 19% as Black, 23% as Asian, and 15% as more than one race. In addition, 12% identified as Hispanic or Latino, while 88% did not. The sample represented a wide range of socioeconomic backgrounds, as measured by parental education and household income. See Table 1 for sociodemographic information. Legal guardians provided informed consent and youth provided assent. All study procedures were approved by the Institutional Review Board at Harvard University. For each monthly visit conducted, participants were compensated approximately $25/hour, amounting to about $12 for completing the assessments in the current study (20 min total).

**Table 1:**
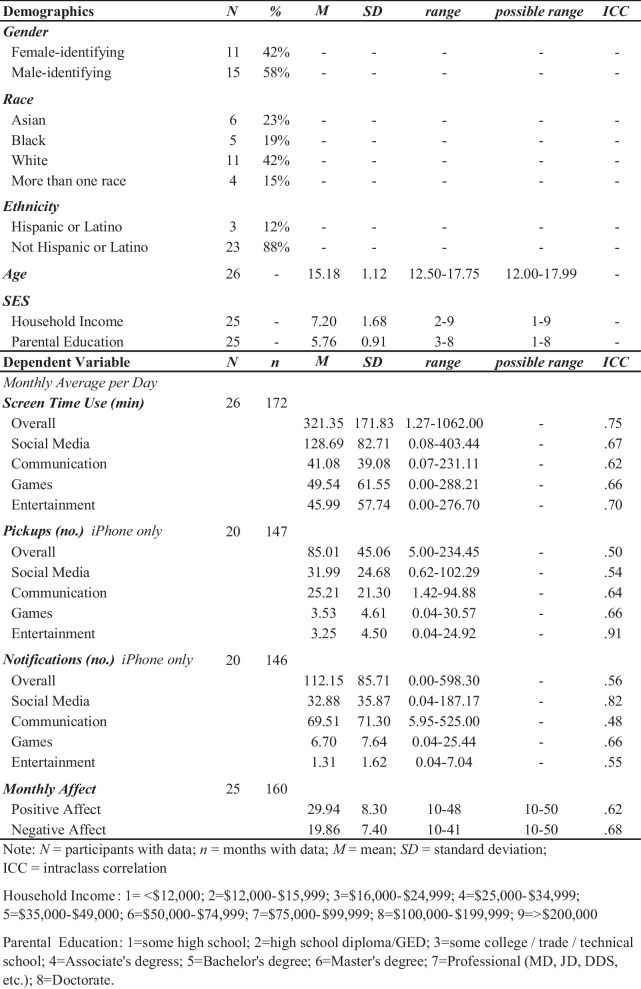
Descriptive summaries

### Procedures

Continuous passive sensing of smartphone use was measured and aggregated to the monthly level. Assessments measuring positive and negative mood were administered at the end of each month. Thus, the structure of the study includes an inherently lagged design between smartphone use and affect measures (i.e., mood) that allow for prospective analyses within the same month and the following month. This monthly timescale is particularly relevant for long-form oscillations in mood and psychological symptoms that tend to occur over weeks or months (Connell & Dishion, 2006; Hammen, 2005). Long-form timescales also have significance for smartphone use and psychological symptoms (Rodman et al., 2021; Thomée et al., 2011), and may identify more cumulative effects that have relevance for clinical risk (Nesi et al., 2022). The current study resulted in a total of 229 possible monthly observations of smartphone use and mood over the study period, with participants completing 182 monthly assessments (80% completion rate).

### Assessments

#### Positive and Negative Affect Scale

The PANAS is a 20-item self-reported measure designed to assess the two primary dimensions of mood, positive affect (PA) and negative affect (NA) (Watson et al., 1988). The PANAS lists 20 feelings, 10 referring to PA (i.e., attentive, interested, alert, excited, enthusiastic, inspired, proud, determined, strong and active) and 10 NA (i.e., distressed, upset, hostile, irritable, scared, afraid, ashamed, guilty, nervous, jittery). Respondents rated the extent to which they have felt each feeling in the past month, using a 5-point Likert scale (from 1 = ‘very slightly or not at all’ to 5 = ‘Extremely’). This scale can be used to test for affect at various timescales, including momentary, daily, weekly, or monthly reports (Watson et al., 1988). Reported affect at longer time scales over weeks or months can be described as mood (Watson et al., 1988). The alpha reliabilities are acceptably high, ranging from .86 to .90 for PA and from .84 to .87 for NA. The PANAS has demonstrated moderate to good reliability in adolescents (.76 positive, .69 negative) (Allan et al., 2015; Crawford & Henry, 2004). See Table 1 for descriptive statistics and intraclass correlation.

#### Smartphone Use

Each month, participants submitted data that indexed their smartphone use. For Android users (*N* = 6), screen time data is retrievable through *MetricWire.* For iOS users (*N* = 20), participants took screenshots of their phone usage via the *Screen Time* report in settings, which displays screen time (i.e., duration), pickups (i.e., first app launched after opening phone), and notifications for all apps on the weekly level going back 1 month. This data was hand-coded and quality checked by team personnel to record the number of minutes spent on each app each week for all participants. All data were calculated on a per day basis (to account for varying number of days each month) and aggregated to the monthly level. Data were then categorized into domains of app type, and we selected both social and non-social categories of use germane to adolescent socioemotional development and wellbeing (Allaby & Shannon, 2020; Marciano et al., 2022): Social Media, Communication, Games, and Entertainment. The Social Media category included apps such as TikTok, Instagram, and Snapchat. The Communication category included apps such as text messaging, FaceTime, WhatsApp, and Phone Calls. The Games category included apps such as Clash of Clans and Two Dot, smartphone-based computer games. The Entertainment category included apps such as Youtube and Netflix. App category definition was defined using guidance from The Apple Store and Google Play (see App Categorization https://osf.io/t36wd/).

### Analytical Approach

We calculated descriptive statistics, correlations, and intra-class correlations (ICC) for all variables (see Table 1 and Table S2). We examined within-person fluctuations in screen time, pickups (iPhone only, Android does not have this metric), and notifications (iPhone only, Android does not have this metric) for various categories of smartphone use (e.g., social media use, communication, games, and entertainment) and examined their prospective, bidirectional associations with monthly measures of positive and negative mood in the same month and the following month (see Table 2). We isolated within-person effects, while controlling for between-person effects. Sensitivity analyses were conducted to account for pickups in notification models and vice versa (Table S1) and found that these variables act as suppressors for one another. Though correlated (maximum zero-order *r* = .500, *p* < .001), these constructs are not completely overlapping (maximum 25% shared variance). Thus, we chose to move forward with their analyses in separate models.

**Table 2:**
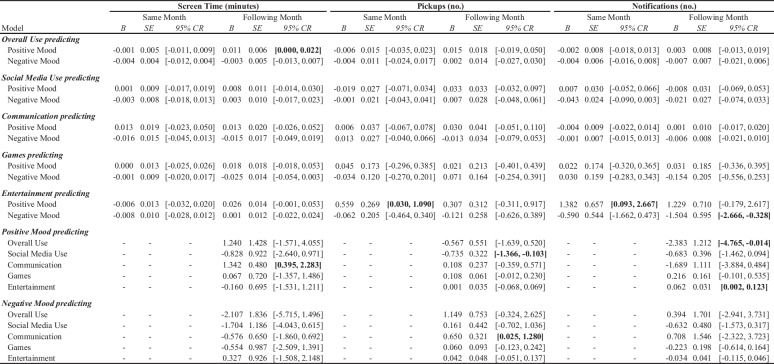
Bayesian hierarchical model outcomes

As in prior work (Rodman et al., 2021), all regression analyses were carried out in a Bayesian framework, due to its appropriateness for exploratory analyses and intuitive interpretation of the 95% highest posterior density (HPD) credible interval (CR), which signifies a 95% probability of the true population parameter being within the interval. We conducted Bayesian hierarchical linear models with unit of time (i.e., study month) nested within subject, with a random intercept allowed to vary across subjects. All models included study month, gender, and age as nuisance covariates. Models were estimated in R 3.5.2 (R Core Team, 2020) using the *Stan* language (Stan Development Team, 2018) and the *brms* (Bürkner, 2017) and *sjstats* packages (Lüdecke, 2019). Weakly informative priors specifying a Gaussian distribution (*M* = 0, *SD* = 10) were used to represent our diffuse prior knowledge of the fixed and random effects. For each parameter, we sampled from 4 stationary Markov chains that approximated the posterior distribution using the Monte Carlo No U-Turn Sampler (Hoffman & Gelman, 2014). Each Markov chain comprised 15,000 sampling iterations, including a burn-in period of 2500 iterations, which were discarded. Convergence of the 4 chains to a single stationary distribution was assessed via the Gelman-Rubin convergence statistic (Gelman & Rubin, 1992). Highest posterior density 95% CR for all parameters was then calculated from these samples and carried forward for inference, wherein CRs that did not contain zero were considered statistically significant.

To dissociate between- and within-person effects of predictors of interest in monthly level analyses, we used within-individual centering (i.e., centering each participant’s observations at the monthly level around their person-specific mean across the year-long study period) and between-subject centering at the year level (i.e., centering each participant’s mean level for the entire study period relative to the overall mean for the entire sample). Both within and between-person terms were included in all models at the same time. This approach orthogonalizes variation in a given predictor into between- and within-person variability (Enders & Tofighi, 2007), accounting for the dependent nature of the data both over time and within-subject, while controlling for trait-level characteristics of each predictor. When assessing within-person effects at the monthly level, we computed both same-month and following-month models to assess for relatively shorter and longer-range prospective relationships.

## Results

### Screen Time

**Fig. 1:**
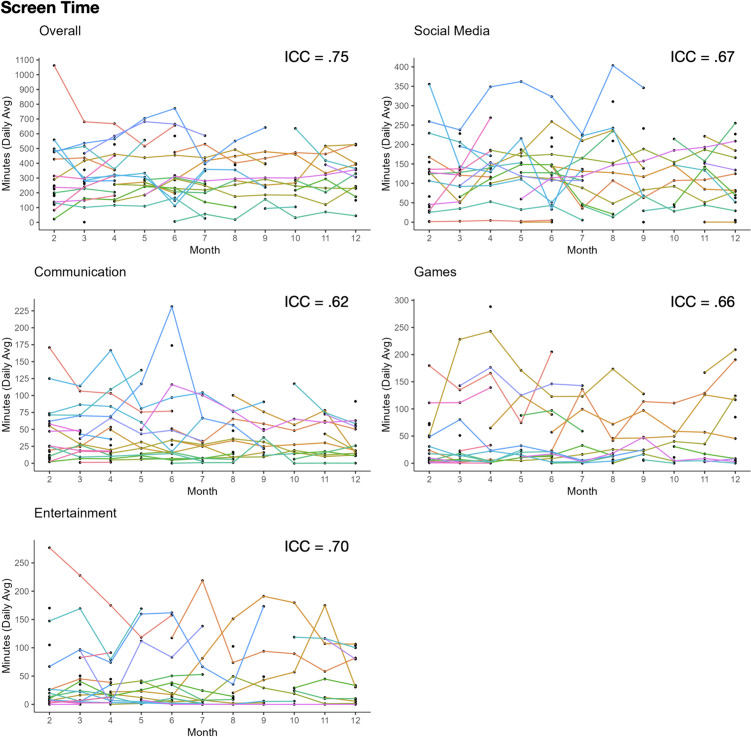
Within-person fluctuations in screen time by category of use

Findings showed substantial within-person variability in screen time for social media, communication, and games (ICCs = .62–.67), and moderate within-person variability for overall use and entertainment (ICCs = .70–.75). On average, adolescents spent 321 min on their phones per day (range: 1.27–1062 m), with the most time engaged in social media for an average of 128.69 min per day (range: 0.08–403.44 m), followed by games (*M* = 49.54 m, 0–288.21 m), entertainment (*M* = 45.99 m, 0–276.70 m), and communication (*M* = 41.08 m, 0.07–231.11 m). See Fig. 1.

#### Mood and Subsequent Screen Time

**Fig. 2:**
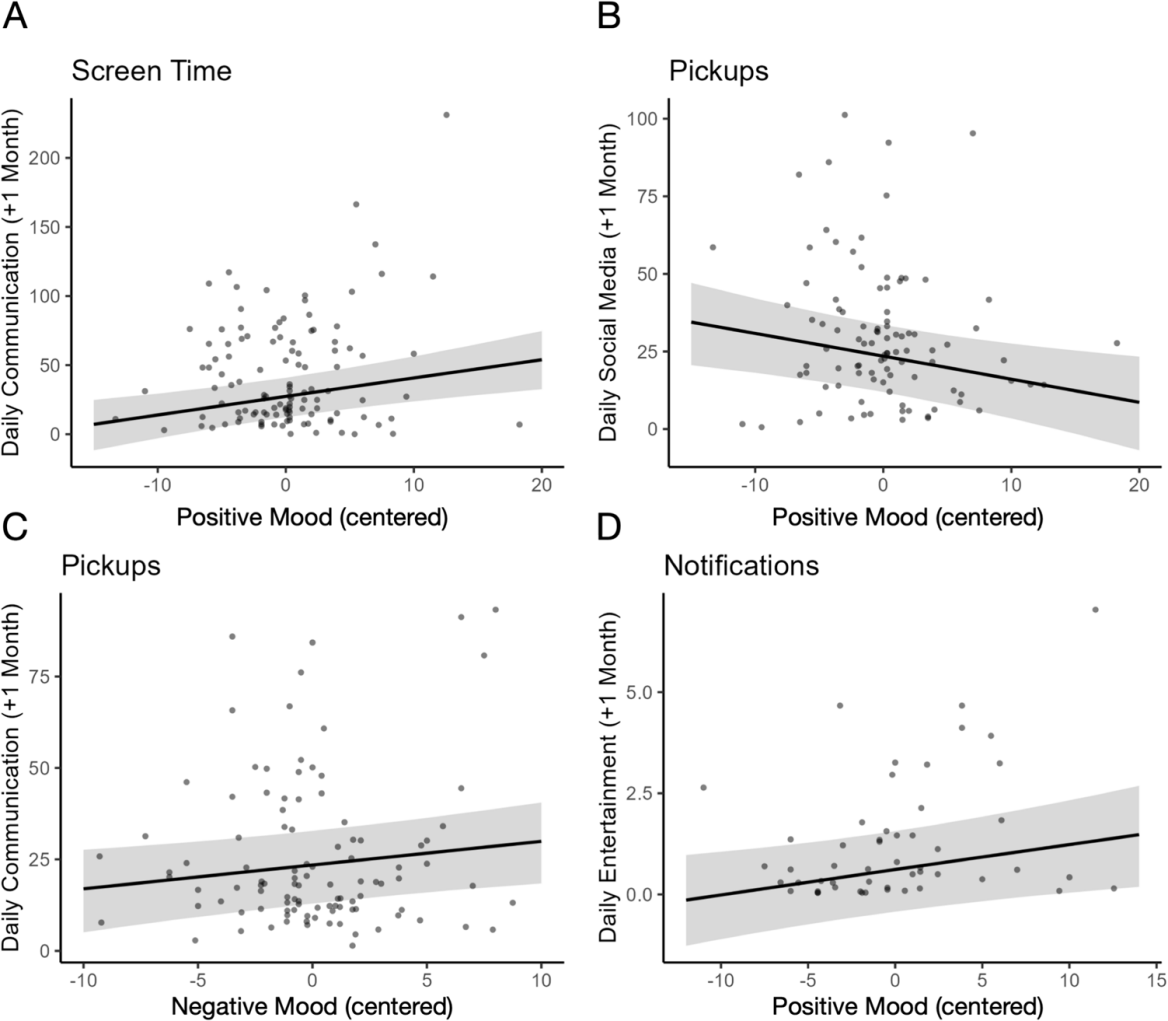
Fluctuations in mood associated with subsequent smartphone use. Improvements in mood (greater positive or less negative) were associated with more screen time on communication apps (A), less pickups for social media (B) and communication apps (C), and more notifications from entertainment apps (D) the following month

Analyses examining the prospective relationships between screen time and mood at the within-person level showed that when adolescents reported greater positive mood than usual, they also engaged in more use of communication apps the following month (*B* = 1.343, *SE* = 0.480, CR = [0.395, 2.283]; Fig. 2A). We did not find other significant associations between fluctuations in positive or negative mood and subsequent changes in other categories of screen time during the following month (CRs included 0). See Table 2.

#### Screen Time and Subsequent Mood

**Fig. 3:**
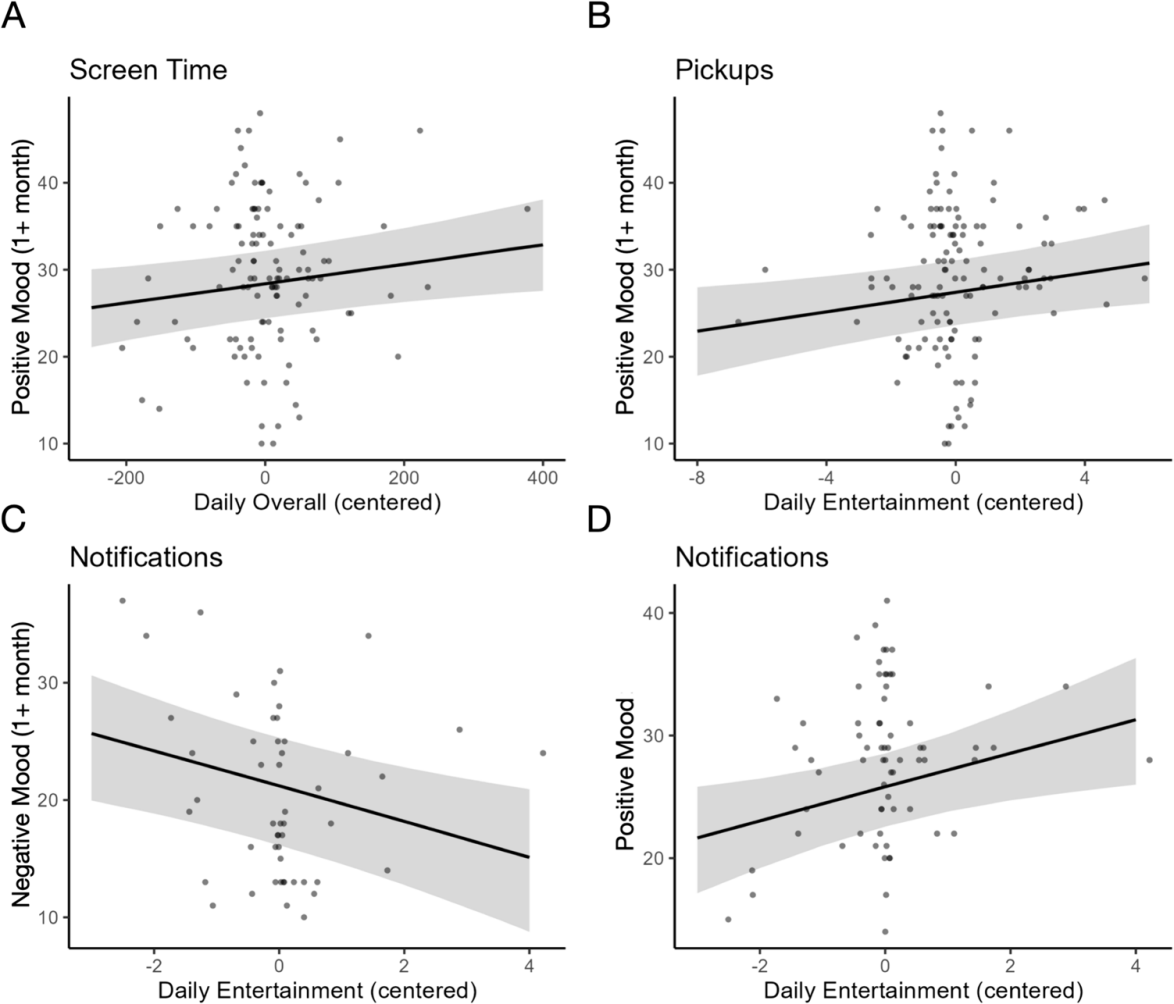
Fluctuations in smartphone use associated with subsequent mood. Greater overall screen time (A), pickups for entertainment apps (B), and notifications for entertainments apps (C, D) were associated with subsequent improvements in mood (greater positive or less negative)

Analyses examining relationships in the other direction showed that when adolescents engaged in more overall screen time than usual, they reported increased positive mood the following month (*B* = 0.011, *SE* = 0.006, CR = [0.001, 0.022]; Fig. 3A). Once again, we did not find other significant associations between fluctuations in other categories of screen time and subsequent changes in positive or negative mood during the same or following month (CRs included 0).

### Pickups

Findings showed substantial within-person variability in overall pickups per day (ICC = .50), with significant within-person variability in pickups for social media, communication, and games (ICCs = .54–.66) and little within-person variability for entertainment (ICC = .91). Overall, adolescents launched apps upon picking up their phones an average of 85 times a day (range: 5.00–234.45 pickups), with pickups for social media apps occurring the most (*M* = 31.99 pickups, range: 0.62–102.29 pickups), followed by communication (*M* = 25.21 pickups, range: 1.42–94.88 pickups), games (*M* = 3.53 pickups, range: 0.04–30.57 pickups), and entertainment (*M* = 3.25 pickups, range: 0.04–24.92 pickups). Pickup analyses were restricted to iPhone users (*N* = 20) because Android does not provide this metric. See Supplementary Fig. 1.

#### Mood and Subsequent Pickups

Analyses examining the prospective relationships between pickups and mood showed that when adolescents reported greater positive mood than usual, they also engaged in fewer pickups of social media apps the following month (*B* = − 0.735, *SE* = 0.322, CR = [− 1.366, − 0.103]; Fig. 2B). Similarly, when adolescents reported less negative mood than usual, they also engaged in fewer pickups of communication apps the following month (*B* = 0.650, *SE* = 0.321, CR = [0.025, 1.280]; Fig. 2C). We did not find other significant associations between fluctuations in positive or negative mood and subsequent changes in other categories of pickups during the following month (CRs included 0). See Table 2.

#### Pickups and Subsequent Mood

Analyses examining relationships in the other direction showed that when adolescents engaged in more pickups of entertainment apps than usual, they reported increased positive mood (*B* = 0.559, *SE* = 0.269, CR = [0.030, 1.090]; Fig. 3B). Once again, we did not find other significant associations between fluctuations in other categories of pickups and subsequent changes in positive or negative mood during the same or following month (CRs included 0).

### Notifications

Findings showed significant within-person variability in notifications overall per day (ICC = .56), with substantial within-person variability in notifications for communication, games, and entertainment apps (ICCs = .48–.66) and moderate within-person variability for social media notifications (ICCs = .82). Overall, adolescents received an average of 112 notifications a day, with the most notifications received from communication apps (*M* = 69.51 notifications, range: 5.95–525.00), followed by social media apps (*M* = 32.88 notifications, range: 0.04–187.17), games (*M* = 6.70 notifications, range: 0.04–25.44) and entertainment (*M* = 1.31 notifications, range: 0.04–7.04). Notification analyses were restricted to iPhone users (*N* = 20) because Android does not provide this metric. See Supplementary Fig. 2.

#### Mood and Subsequent Notifications

Analyses examining the prospective relationships between notifications and mood showed that when adolescents reported greater positive mood than usual, they also received fewer notifications overall (*B* = 2.383, *SE* = 1.212, CR = [− 4.765, − 0.014] and more notifications from entertainment apps the following month (*B* = 0.062, *SE* = 0.031, CR = [0.002, 0.123]; Fig. 2D). We did not find other significant associations between fluctuations in positive or negative mood and subsequent changes in other categories of notifications during the following month (CRs included 0). See Table 2.

#### Notifications and Subsequent Mood

Analyses examining relationships in the other direction showed that when adolescents received more notifications from entertainment apps than usual, they reported increased positive mood the same month (*B* = 1.382, *SE* = 0.657, CR = [0.093, 2.667]; Fig. 3C) and decreased negative mood the following month (*B* = − 1.504, *SE* = 0.595, CR = [− 2.666, − 0.328]; Fig. 3D). We did not find other significant associations between fluctuations in other categories of notifications and subsequent changes in positive or negative mood during the same or following month (CRs included 0).

## Discussion

The current study used passive sensing data to extract smartphone use across multiple metrics (screen time, pickups, notifications) and categories (overall, social media, communication, games, and entertainment) and investigated their prospective, bidirectional relationships with monthly measures of positive and negative mood over a year. On average, adolescents used their smartphones for about 5 h a day and showed substantial within-person variation (~ 40%). When adolescents reported better mood than usual, they subsequently used communication apps more and checked social media and communication apps less. Meanwhile, when adolescents engaged in more use of entertainment apps than usual across all metrics, they subsequently reported improved mood. In all, these findings preliminarily suggest a pattern where fluctuations in mood relate to subsequent changes in smartphone behavior that were primarily in the social domain, whereas fluctuations in smartphone behavior predicting subsequent changes in mood were primarily in the entertainment domain. We found little evidence that within-person fluctuations in screen time or social media use were associated with increases in negative mood, as frequently theorized. These findings highlight the importance of disentangling the distinct components of smartphone use that relate to affective processes and examining their bidirectional, prospective relationships over time, due to the possibility of differential outcomes.

Passive sensing data allowed for the characterization of how teens use their phones across various metrics and categories over time. The data revealed that smartphone use fluctuates substantially from month to month, whereby 30–38% of the variance is driven by within-person variability in screen time, 34–50% in pickups (except entertainment at 8%), and 18–44% in notifications, which is in line with prior work using objective measures (Bradley & Howard, 2023; Harari et al., 2019; Rodman et al., 2021). Average smartphone use in the present study is also comparable to other studies using objective smartphone data finding average daily screen time of 3–8 h (Andrews et al., 2015; Bradley & Howard, 2023; Ellis et al., 2019; Sewall et al., 2020), and around 100 pickups and notifications a day (Bradley & Howard, 2023; Ellis et al., 2019), with most occurring for social apps (Bradley & Howard, 2023). Importantly, the descriptive statistics and ICCs from subjective measures of smartphone use differ markedly from the type of objective measures used here. ICCs of self-reported smartphone use are substantially lower than objective measures (ICCs ~ .15–.30 lower), (Coyne et al., 2020; Marciano et al., 2022) which may, in part, reflect bias in subjective reports (Boyle et al., 2022; Sewall et al., 2020) and underscores the need for objective measurement of smartphone use. Indeed, self-reported screen time tends to be underestimated and social media app use tends to be overestimated relative to objective metrics (Andrews et al., 2015; Boyle et al., 2022; Parry et al., 2021; Sewall et al., 2020). Furthermore, adolescent smartphone use fluctuates substantially from month to month, which highlights the need to consider within-person (vs. between-person) associations between smartphone use and affective processes.

We examined the prospective, bidirectional relationships between smartphone use (across varied categories and metrics) and mood. Findings revealed a weakly positive relationship between greater overall screen time than usual and subsequent increased positive mood. While subjective reports of phone use are primarily related to worse wellbeing or mood (Anderl et al., 2023; Sewall et al., 2020), actual phone use often shows small or non-existent associations with mood (Marciano et al., 2022; Sewall et al., 2020; Shaw et al., 2020). When examining the reverse direction, adolescents who reported greater positive mood than usual showed greater subsequent screen time on communication apps. Prior work has also shown that positive mood relates to subsequent increases in smartphone use (Marciano et al., 2022), and it stands to reason that the enhanced sociability that follows improved mood in real life (Whelan & Zelenski, 2012) would extend to social smartphone use. When examining pickup behavior (app launched after pickup), launching entertainment apps more than usual was associated with subsequent increases in positive mood, in line with prior work showing online leisure activities relate to positive affect (Stoeber et al., 2011). By contrast, better mood than usual was associated with subsequent decreases in pickups for social media and communication apps. Pickups of social apps are often conceptualized as *checking* behavior that reflects an updating process within the context of social communication or milieu. Indeed, the majority of pickups are related to social media checking (Bradley & Howard, 2023) and have been associated with negative mood (Saeb et al., 2015). Finally, when adolescents received more notifications from entertainment apps than usual, they reported better subsequent mood. While more data is needed to parse meaningful contextual factors, notifications from entertainment apps may reflect other participant behaviors (e.g., leisure time; Fennell et al., 2019). Indeed, increased independence during adolescence is accompanied by increased leisure time (Larson, 2001). Smartphone use is highly shaped by availability, where adolescents check social apps when free for short durations, but when available for longer durations, they watch shows and videos (Toh et al., 2019). Leisure time has been associated with improved mood and reduced stress (Zawadzki et al., 2015), and leisure-time smartphone use was found to be a protective factor against depression (Kremer et al., 2014). Thus, notifications from entertainment apps may reflect relaxed scheduling restraints that, in turn, relate to improved mood.

Taken together, the current findings illustrate a pattern where the directionality of influence between smartphone use and mood may depend on the category of phone use, such that fluctuations in mood appear to influence socially related smartphone behaviors, and smartphone use that is related to subsequent changes in mood is primarily entertainment-related (perhaps, as a marker of leisure time). While this differential pattern must be replicated, it preliminarily demonstrates the importance of examining bidirectional relationships in a longitudinal design. Much extant research examining smartphone use and adolescent wellbeing is based on cross-sectional studies testing between-person differences at a single point in time (Boer et al., 2020; Liu et al., 2022; Shannon et al., 2022; Twenge et al., 2018) and these designs cannot capture the substantial within-person variability evident in smartphone use (Beyens et al., 2021; Valkenburg et al., 2022). Moreover, repeated sampling designs permit the examination of bidirectional relationships, where wellbeing can shape subsequent smartphone behaviors (Katevas et al., 2018; Rodman et al., 2021) or clarify relationships that exist in one direction only (Anderl et al., 2023; Marciano et al., 2022). Therefore, examination of bidirectional, prospective relationships, which is possible in a high-frequency longitudinal design as in the current study, is crucial to observe how these relationships unfold over time (Orben, 2020).

Critically, very weak or no associations were found with overall phone use, and patterns primarily emerged when examining more granular features of smartphone use. Prior work supports this finding, wherein overall trends in smartphone use and wellbeing were further qualified by specific types of platform and metrics of phones use (David et al., 2018; Marciano et al., 2022; Oulasvirta et al., 2012; Rozgonjuk et al., 2018). Presently, most research examining smartphone use and adolescent functioning has largely taken a monolithic approach focused on total screen time as a basis for extrapolating outcomes (Mougharbel & Goldfield, 2020; Nesi & Prinstein, 2015). This may obfuscate meaningful relationships, as adolescents use smartphones in a multifarious manner, all with varying effects (Toh et al., 2019; Twenge & Farley, 2021). Thus, understanding how smartphone use impacts adolescent wellbeing necessitates measures that make meaningful distinctions between platforms, features, or uses (Prinstein et al., 2020).

### Limitations and Future Directions

Given that adolescence is a period of social and affective development that is accompanied by risk for mental health problems, it is critical to improve measurements to examine how smartphone use relates to wellbeing (Orben et al., 2024). In this study, we use objective smartphone measures across multiple categories of phone use and utilize a longitudinal design to examine within-person, bidirectional relationships. However, the current findings have several limitations. While the study is powered to examine these questions at the within-person level (see *Supplementary Information*), the current sample is small and may not generalize; however, the range in gender, racial/ethnic, and socioeconomic representation helps to mitigate some of these concerns (Table 1). Furthermore, the sample size did not permit examination of between-person or age-related effects, which are an important consideration (Beyens et al., 2021; George et al., 2018; Orben et al., 2022). While the current study focuses on monthly fluctuations that capture long-form oscillations in variables of interest, future work should also include examination of short-form dynamic oscillations on the within-day or daily level. Additionally, participants were freely able to use other devices, put time limits on their screen time or customize the silencing of their notifications. While we aimed to capture naturalistic behavior, this may have inadvertently introduced some noise to our data, driving down metrics of smartphone use in ways that may systematically vary by category. Future work should account for such participant-set stipulations and behaviors in analyses. Finally, a future work should combine quantitative and qualitative approaches to further understand the motivations and functions of smartphone use.

## Conclusion

The current study aimed to leverage intensive, longitudinal sampling of objective smartphone data across multiple platforms (i.e., app types) and metrics to characterize the prospective, bidirectional associations between fluctuations in smartphone use and adolescent mood over time. We found little evidence that within-person fluctuations in screen time or social media use were associated with increases in negative mood, as frequently theorized. Instead, when differentiating between various smartphone features, preliminary patterns emerged: fluctuations in mood related to subsequent changes in smartphone use that are primarily social, whereas fluctuations in smartphone use related to subsequent changes in mood were primarily entertainment-related. This work highlights the need to make meaningful distinctions between platforms, features, or uses as a necessary first step in identifying targets for intervention efforts to promote resilience and wellbeing during adolescence.

## Supplementary Information

Below is the link to the electronic supplementary material.

Supplementary file1 (PDF 248 KB)

## **Additional Information**

### Data Availability

All data and code are made available on Open Science Framework at: https://osf.io/t36wd/.
