# Supplementary material for: Within-Person Fluctuations in Objective Smartphone Use and Emotional Processes During Adolescence: An Intensive Longitudinal Study
Source: Affect Sci. 2024 Jul 24;5(4):332–45. doi: 10.1007/s42761-024-00247-z (PMC11624186; doi:10.1007/s42761-024-00247-z)
Supplement: Supplementary file 1 — Supplementary file1 (PDF 248 KB) [file 42761_2024_247_MOESM1_ESM.docx]

Supplemental Materials

to accompany

Within-person fluctuations in objective smartphone use and emotional processes during adolescence: An intensive longitudinal study

Rodman, Burns, Cotter, Ohashi, Rich & McLaughlin

For *Affective Science Special Issue: Affect and Social Media*

**Supplemental Materials**

**Simulation Power Analysis**

To test our ability to detect within-person effects (the focus of our study), we conducted statistical power simulations based on the distribution, mean, and dispersion of our variables of interest. We tested statistical power to detect small (standardized beta = 0.12), medium (standardized beta = 0.3), and large effect sizes (standardized beta = 0.5) for associations between smartphone use and wellbeing. Our simulations assumed 11 observations per participant and evaluated sample sizes from 10 to 50 participants. Results revealed that with 26 participants (11 observations each; collection of smartphone data began *after* the first visit) we were well-powered (>80%) to detect small, medium, and large within-person effects for our variables of interest (Figure S3). It is important to note that while all analyses presented in our manuscript were conducted using a Bayesian approach, our power analyses were conducted using a standard frequentist approach, given that statisticians have not come to a consensus regarding the meaning of power in a Bayesian context (in Bayesian analysis all the focus is on estimation and not on testing), and much less regarding a standard way of computing statistical power akin to the frequentist way commonly used in psychology studies.

**Supplemental Figures**

**
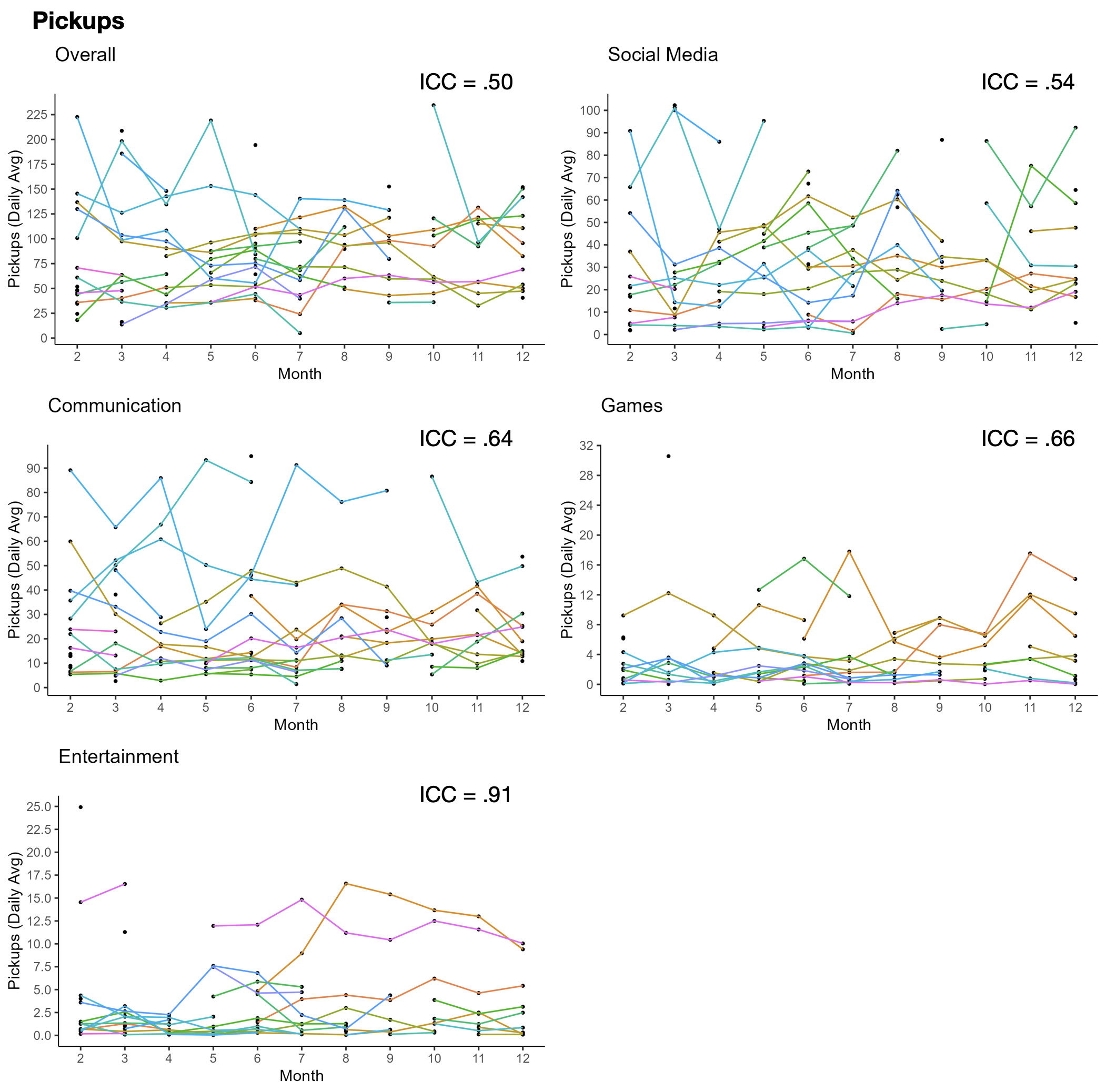
**

Figure S1. Within-person fluctuations in pickups by category of use.


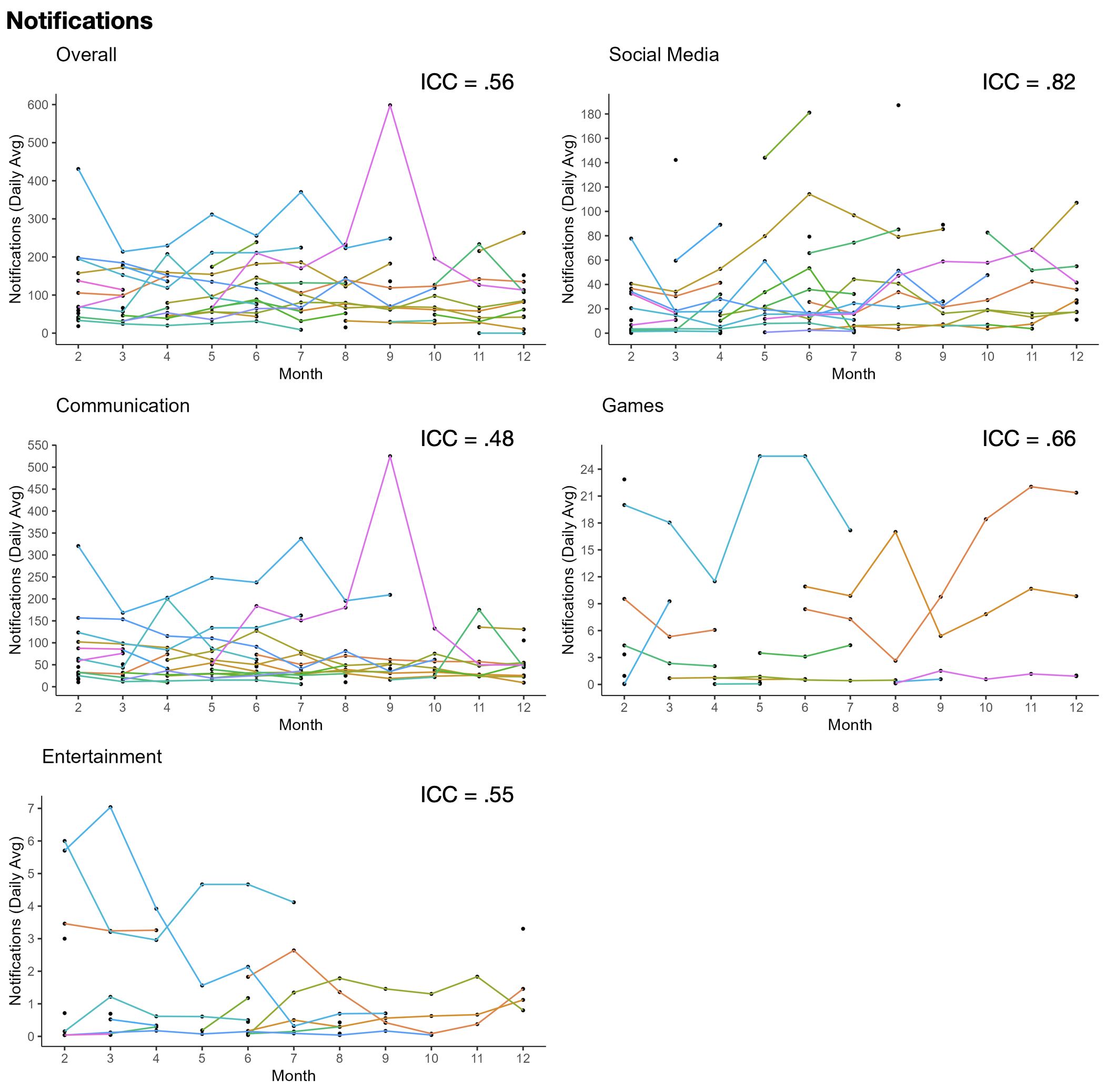


Figure S2. Within-person fluctuations in notifications by category of use.


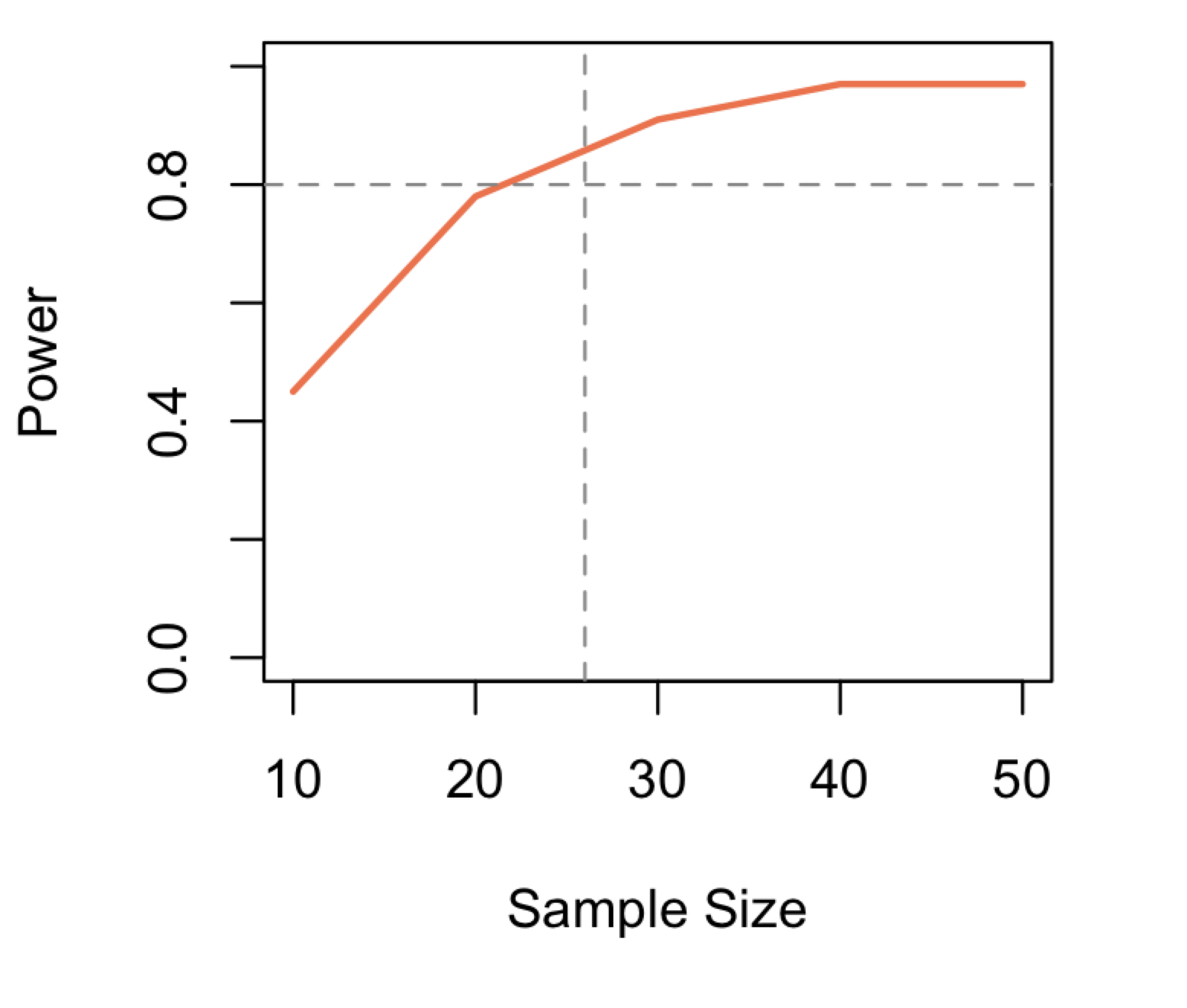


Figure S3. Power to detect within-person effects.

**Supplemental Tables**
